# Prioritizing vaccination based on analysis of community networks

**DOI:** 10.1007/s41109-022-00522-7

**Published:** 2022-12-02

**Authors:** Katherine Klise, Walt Beyeler, Erin Acquesta, Haedi Thelen, Monear Makvandi, Patrick Finley

**Affiliations:** Sandia National Laboratories, Albuquerque, NM US

**Keywords:** Vaccination, Community demographics, Network analysis, Global sensitivity analysis, COVID-19

## Abstract

Many countries that had early access to COVID-19 vaccines implemented vaccination strategies that prioritized health care workers and the elderly. As barriers to access eased, vaccine prioritization strategies have been relaxed. However, these strategies are still an important tool for decision makers to manage new variants, plan for future booster shots, or stage mass vaccinations. This paper explores the impact of vaccine prioritization strategies using networks that represent communities with different demographics and connectivity. The impact of vaccination is compared to non-medical intervention to reduce transmission. Several sources of uncertainty are considered, including vaccine willingness and mask effectiveness. This paper finds that while prioritization strategies can have a large impact on reducing deaths and peak hospitalization, selecting the best strategy depends on community characteristics and the desired objective. Additionally, in some cases random vaccination performs as well as more targeted prioritization strategies. Understanding these trade-offs is important when planning vaccine distribution.

## Introduction

While access to COVID-19 vaccines is still lacking in many parts of the world, vaccine distribution is well underway in many middle- to high-income countries [1]. Given that vaccine supplies were initially limited, vaccine prioritization strategies were created to maximize health benefits and mitigate health inequities [2, 3]. These strategies commonly include giving vaccine priority to health care workers and the elderly. As barriers to access started to ease and vaccines were approved for younger age groups, vaccine prioritization strategies have been relaxed. However, even with a majority of the population in the United States eligible for vaccines, there are significant challenges to overcome before the population reaches herd immunity. For example, the uptick in cases associated with the Delta and Omicron variants illustrates remaining risks where vaccination rates are low [4]. In the absence of herd immunity, it is important to maximize the impact of vaccination. Furthermore, uncertainty in the strength and duration of immunity, especially in light of recent variants, highlights the importance of developing prioritization strategies for future vaccination.

Vaccine prioritization strategies have been the subject of numerous recent research studies, many of which use analysis based on susceptible-exposed-infected-recovered (SEIR) models. The modeling analysis conducted by Bubar et al. [5] used an age stratified model with variable contact rates, along with variability in vaccine efficacy, susceptibility, seropositive status, and speed and timing of vaccine rollout to track cumulative incidence, mortality, and years of life lost. They concluded that vaccination of older adults is near optimal considering plausible vaccine characteristics. Ferranna et al. [6] also used an age stratified model to study vaccine prioritization. The study includes vaccine allocation based on age and essential workers and tracks avoided deaths, avoided infections, and life years gained. They also conclude that prioritizing older adults has the best outcomes, and high equity weights are required to prioritize essential workers over the elderly. Tetteh et al. [7] used random graphs to model the impact of mass vaccination as compared to ring vaccination, which targets individuals in contact with confirmed cases and was instrumental in the elimination of Smallpox [8]. The analysis concluded that ring vaccination could be effective at lowering the total number of infections, but requires rigorous contact tracing. Antonopoulos et al. [9] use network models based on random, small-world, and scale-free structure to study interconnected communities with different levels of vaccination and find detrimental effects from non-vaccinated communities on vaccinated communities. Yang et al. [10] used network models based on survey data from several communities in India to study the impact of vaccination strategies. This work used sampling methods that reveal various amounts of information on the true network structure given that network metrics such as highest-degree and centrality are rarely known in actual populations. Results show that prioritization strategies can improve epidemic outcomes even when networks are only partially observed. Chapman et al. [11] study the impact of vaccine prioritization strategies across California using multiple risk factors based on age, location, occupation and other classification. Results show that the use of multiple risk factor can improve prioritization and that results vary by county.

This paper builds on previous work to create a modeling framework that compares the impact of vaccination and non-pharmaceutical interventions using networks that represent specific communities at the county scale. The network representations of each community includes heterogeneous structure with respect to age, household size, and contact intensity between individuals. While community transmission is governed by additional factors, such as contact between people at places of business, the network generation methods used here provide a way to generate networks from readily available data. The network representation is coupled with an SEIR based epidemiological model that includes vaccination and non-pharmaceutical interventions [12]. Two-dose vaccines are modeled with two levels of efficacy and a three-week delay between doses. This coupling of network and epidemiological model facilitates a detailed representation of how members of different age groups interact and have different disease risk factors. For example, older individuals are more susceptible to serious disease outcomes but generally have lower level of contacts with other members of the community. This modeling framework is used to compare vaccine prioritization strategies, including random mass vaccination, and vaccination prioritized by age, number of contacts, household size, and contact with known cases (i.e. ring vaccination).

The objective of this paper is to explore the impact of vaccine prioritization strategies using networks that represent different demographics and connectivity and compare those outcomes to non-medical intervention. Several factors influencing disease progression are taken into account, including willingness to be vaccinated, the use of masks, and contact between and within age groups with different levels of social vulnerability. Sensitivity analyses are used to discover the effects of these uncertain factors on prioritization objectives. Understanding these impacts is especially important for decision makers when managing new COVID-19 variants and planning for future booster shots or mass vaccinations in different communities. The paper is organized as follows: Section 2 includes a description of the community networks, disease transmission model, and sensitivity analysis, Section 3 includes simulation results and sensitivity indices, and Section 4 presents a discussion of the findings.

## Methods

The following section describes the community network models and disease transmission model used in this analysis along with parameters and metrics included in a global sensitivity analysis.

### Community networks

Disease spread depends on the way in which members of a community interact. Different interaction patterns may lead to differences in a community’s response to vaccination strategies. For this reason, four communities of interest were identified for this analysis using census and health data at the county level based on several factors, including Social Vulnerability Index (SVI), age distribution, household size distribution, and population. SVI is a measure of potential negative effects on communities caused by stressors such as natural disasters and disease outbreaks, based on 15 social factors, including poverty, transportation, and housing. The data was obtained from the Centers for Disease Control and Prevention (CDC) [13]. Age, household size, and population data was obtained from U.S. census data through SafeGraph [14].

**Table 1:** Communities based on U.S. counties having high SVI, high average age, high average household size, or high population (criteria for selection shown in bold)

Community	County, state	Average age (yr)	Population (people)	SVI (unitless)	Average household size (people)
High age	Sumter, FL	**58.557**	113,589	0.236	1.894
High population	Los Angeles, CA	37.400	**10,057,155**	0.768	2.754
High SVI	Duval, TX	38.616	11,510	**0.999**	2.746
High household size	Oglala Lakota, SD	29.299	14,263	0.994	**3.863**

Communities with high SVI, high average age, high average household size, and high population were selected for the analysis. The communities of interest have the following characteristics: 1) high SVI, based on data from Duval County, Texas, 2) high average age, based on data from Sumter County, Florida, 3) high average household size, based on data from Oglala Lakota County, South Dakota, and 4) high population, based on data from Los Angeles, California. Table 1 includes the SVI, average age, average household size, and population for all four communities. Throughout this paper, communities are listed in the order shown in Table 1, which reflects the level of connectivity and clustering in each network as described later in the paper.

Networks for each community were generated using a two step process that integrates the FARZ network generation algorithm [15] with additional household clusters. Methods to generate the community networks are available in the seirsplus Python package [16]. Similar methods have been used to build community networks that represent interactions at school to study the impact of school reopening during COVID-19 [17]. In this application, the network structure is controlled by the population’s age distribution, household size distribution, and contact within and between age groups. Network nodes represent individuals in the community and the network links represent contacts between individuals. Contact networks can also be generated from other data sources, including contact tracing [18], statistical methods [19, 20] and mobility data [21, 22].

**Fig. 1:**
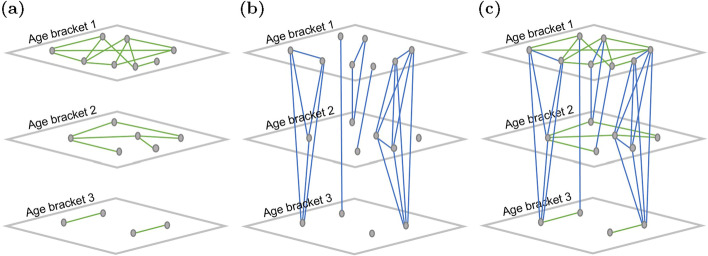
Illustration of the network generation process, including **a** FARZ network for each age bracket, **b** household clusters, and **c** the combined network. Gray nodes represent people, green and blue edges indicate connectivity between people. Age bracket 1 has 8 people with an average node degree of 5, age bracket 2 has 6 people with an average node degree of 4, age bracket 3 has 4 people with an average node degree of 3. There are 7 households, with an average household size of 2.6 (2 households contain 1 person)

The process used to build community networks is illustrated in Fig. 1 and described below. Community networks were generated by first creating separate networks using the FARZ network generation algorithm for people in different age brackets, as shown in Fig. 1a. The FARZ algorithm expands the network one node at a time, using a probability of community assignment proportional to the current community size. The connectivity of these networks is defined by several parameters, including the number of people in each age bracket and the average number of people an individual comes into contact with (the average node degree). The resulting networks have a heavy tailed node degree and community size distribution noted in real communities. The following input parameters are used to generate the FARZ networks (with notation from [15]): 1) the number of people in the age bracket (*n*), 2) the average number of connections between individuals (2*m), defined as the average node degree for the age bracket minus the average household size, and 3) the number of communities (*k*), defined as 1 community for every 50 people. Additional parameters and default values (from [16]) include a clustering parameter ($\alpha$ = 2), assortative parameter ($\gamma$ = −0.6), the probability of membership within a community ($\beta$ = 0.6), and the maximum number of communities each person belongs to (*r*= 1). The communities have a power law size distribution ($\phi$ = 1). These input parameters could be adjusted if desired.

The individual networks are then linked together to account for people that live within the same household (and often span multiple age brackets), as shown in Fig. 1b. Household clusters are created based on the following information: 1) household size distribution and 2) household age characteristics which includes the percent of households that have at least one person under 20, the percent of households that have at least one person over 60, the percent of households that include at least one person under 20 and over 60, the percent of households that are single-occupant with a person over 60, and the average number of children per household. These statistics are used to assign individuals to households. Additional edges are then added to the network to connect individuals from the same household. Members within the same household form fully connected groups, while retaining connectivity to the FARZ network for their age bracket, as shown in Fig. 1c. An iterative approach is used to create a network that meets target statistics within an error tolerance.

Note that the network generation methods do not account for interactions between people in different age groups outside households. For this reason, in the disease transmission model described below, only 90% of transmission is governed by the network structure (meaning that exposure is directly related to a neighboring node being able to transmit the disease). The remaining 10% of transmission occurs randomly, driven by the total number of nodes able to transmit the disease. Future research could adjust this threshold or add additional transmission pathways to reflect structured interactions outside the home.

**Fig. 2:**
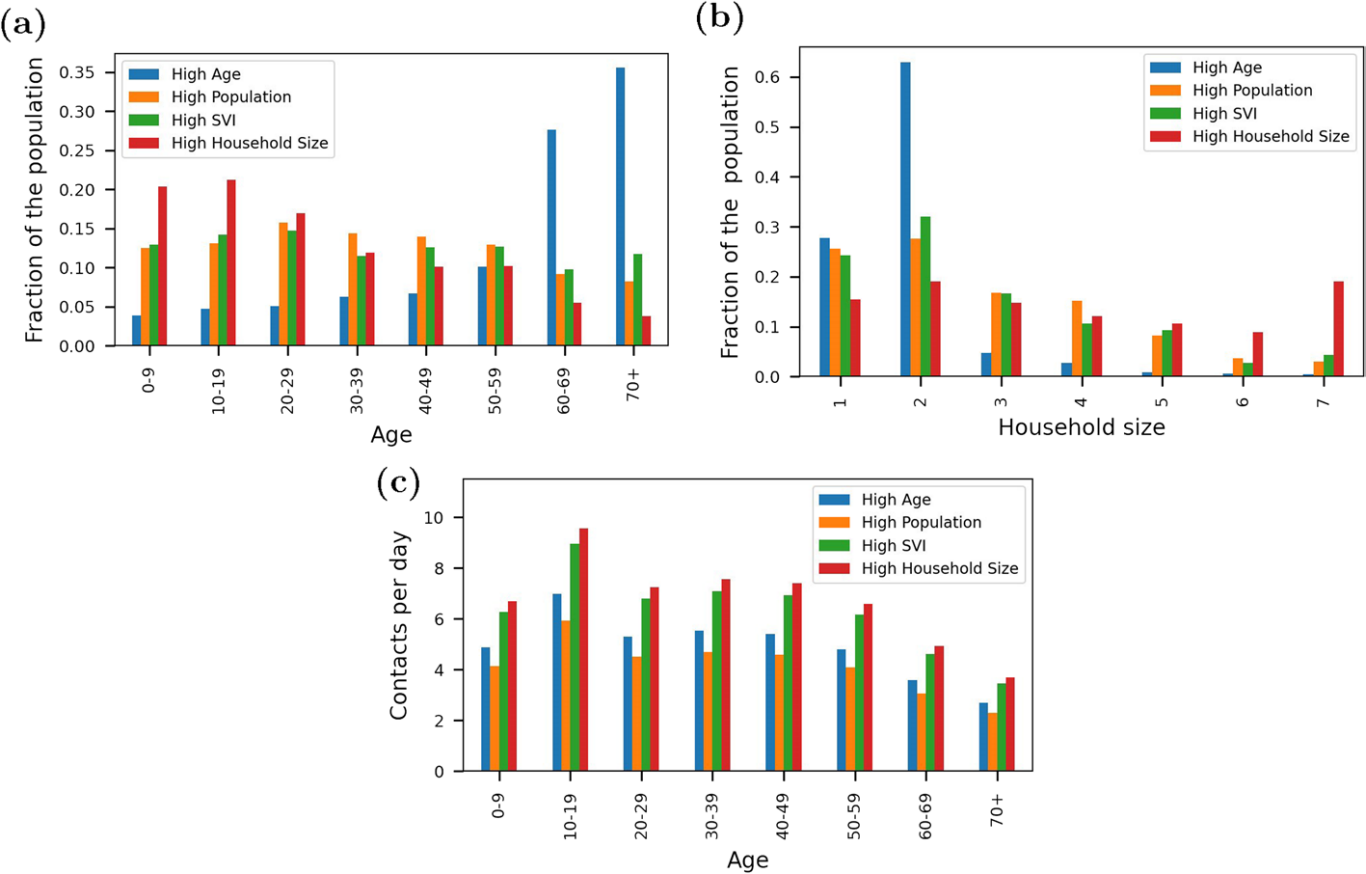
Distribution of parameters used to define community networks including **a** age **b** household size and **c** contacts per day

Community networks were generated for this analysis using the age distribution, household size distribution, and social contact characteristics for each community as shown in Fig. 2. Age and household size distributions for each community were obtained from census data provided by SafeGraph [14]. The data provides a distribution of ages in 10 year increments and groups people into households of 1 person through 7 or more people. The network generation methods also use household age characteristics which are obtained from census data for each county. This includes the percent of households that have at least one person under 20, percent of households that have at least one person over 60, percent of households that include at least one person under 20 and over 60, percent of households that are single-occupant with a person over 60, and the average number of children per household.

Social contacts within age groups were based on a 2008 study by Mossong et al. [23]. The social contacts data has an average of 13 contacts per day. Given how sharply COVID-19 cut social interactions, the values were reduced by 50% in each age group and then further modified to reflect the percent of the population staying at home in each community. The fraction of each population staying at home was obtained from SafeGraph based on mobility data from September 13, 2020 [24]. Because this date is not associated with peak case loads, it is assumed to be representative of contact patterns during the pandemic over a sustained period of time. Note that Oglala Lakota County was not included in the SafeGraph database for this metric and an average value from surrounding counties was used for that community.

**Table 2:** Contact modifier for each community based on the fraction of each population staying at home

Community	County, state	Staying home (fraction)	Difference from baseline (unitless)	Contact modifier (unitless)
High age	Sumter, FL	0.333	0.22	0.39
High population	Los Angeles, CA	0.365	0.34	0.33
High SVI	Duval, TX	0.273	0	0.50
High household size	Oglala Lakota, SD	0.254	−0.07	0.53

While many factors determine one’s ability to stay at home, SVI is a large contributing factor [25]. In this analysis, communities with high SVI (Duval County and Oglala Lakota County) have a relatively low fraction of the population staying home, while communities with low to moderate SVI (Sumter County and Los Angeles County) have more people staying at home. Therefore, a simple “contact modifier” was developed to include the influence of SVI on contact intensity in the network construction. The contact modifier was computed for each community based on an assumed 50% decrease in contacts and a “difference from baseline” using the fraction of people staying home. Duval County, having the highest SVI, was used as the baseline in this analysis and has an assumed contact modifier of 0.5. The contact modifiers for the other three counties were based on their staying home data compared to Duval County. For example, Los Angeles County has 36.5% of the population staying at home while Duval County has 27.3% of the population staying at home. The difference from baseline for Los Angeles County is computed as (0.365 - 0.273)/0.273 = 0.34 and the contact modifier is computed as 0.5*(1-0.34) = 0.33. The contact modifier is used to reduce the number of contacts each day within each age group (as shown in Fig. 2c). Table 2 includes the fraction of people staying home, a difference from baseline, and the contact modifier for each community. More research is needed to define contact intensity in different communities based on demographics and other factors.

It is important to note that all networks are constructed using 5000 nodes even though the total populations in the four communities differ. The disease transmission model uses a representative subset of each community. For example, the High Population community is a representative sample of 5000 individuals based on the age, household, and contact distributions for that community. For each community, multiple realizations of their contact network are generated using a unique seed, which is used to populate the household clusters, age of people within each household, and therefore the contact potential between individuals.

**Fig. 3:**
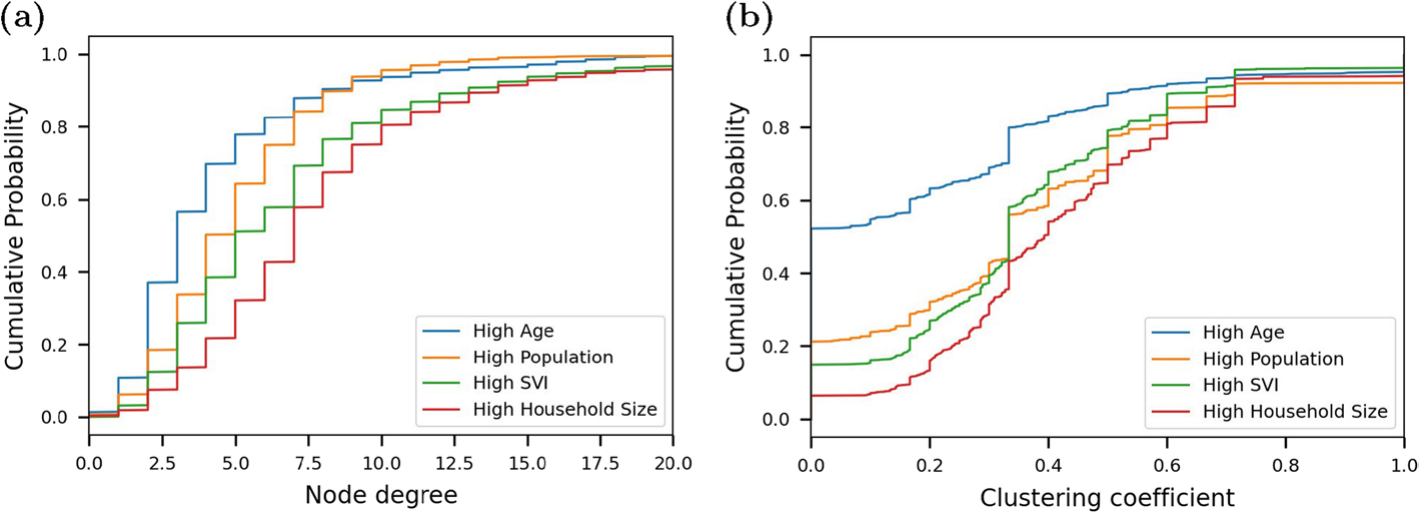
Empirical cumulative distribution for **a** node degree and **b** clustering coefficient from a single realization of each community

Once constructed, the networks store several node attributes, including the number of contacts, age, household size, vaccine status, and disease state of each representative person. These attributes are used to model different vaccine strategies as described in the next section. Two topographic metrics are used to illustrate key differences between community structures that are a result of the network parameterization: average node degree and clustering coefficient. Node degree is defined as the number of neighboring nodes for each node and represents the number of individuals potentially involved in disease transmission. The clustering coefficient is a measure of the degree to which nodes tend to cluster using the number of connections between neighboring nodes. The node degree and clustering coefficient distribution from a single realization of each network is shown in Fig. 3. Additional realizations show similar distributions. Note that the High Age community has the lowest median node degree and clustering coefficient. High Population and High SVI have moderate median node degree and clustering. The High Household Size community has the highest median degree and clustering. Based on these topographic metrics, the High Age community is described as the most isolated while the High Household Size community is described as the most connected.

**Fig. 4:**
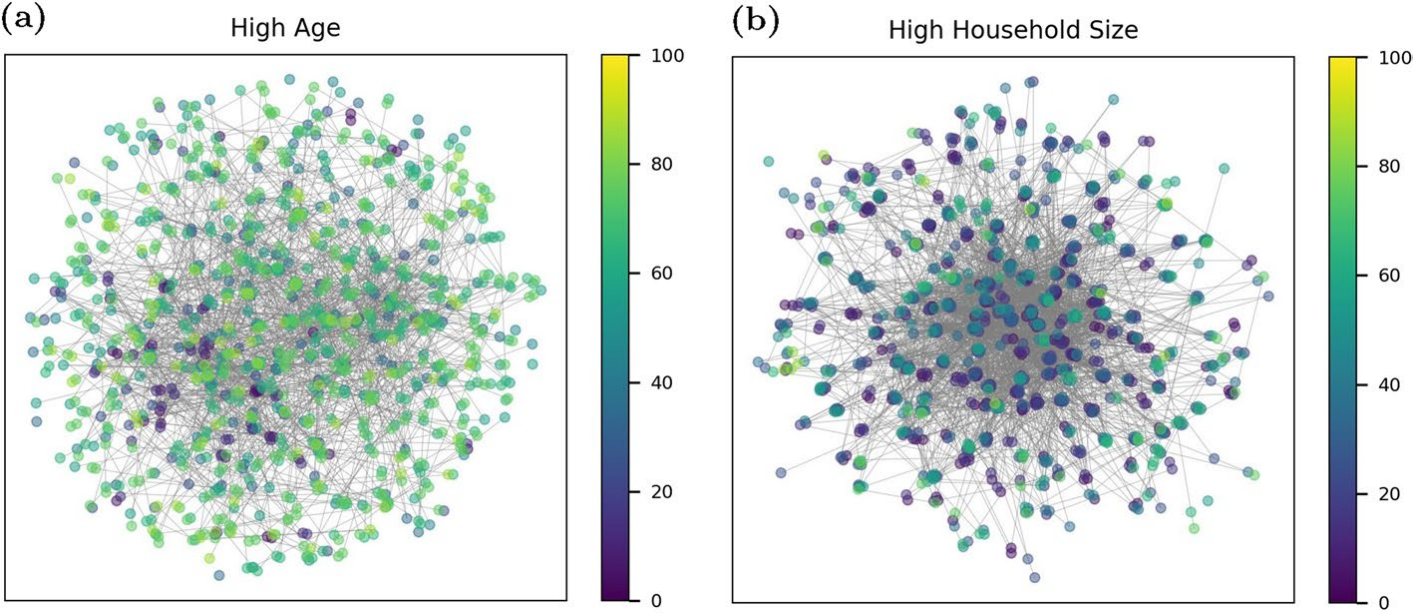
Example community network realization colored by node age for **a** High Age community, and **b** High Household Size community

For illustrative purposes, smaller example networks with 1000 nodes are shown in Fig. 4 to illustrate differences in age distribution, clustering, and connectivity using a High Age and High Household Size community. The node positions are generated using a spring layout to avoid overlap and cluster nodes that are in the same household. Node locations are only used for visualization and are not a factor in the disease transmission model.

### Disease transmission model

**Fig. 5:**
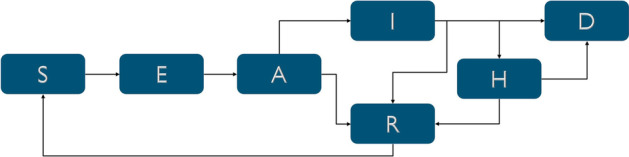
Disease transmission model states and pathways

To study the impact of vaccine prioritization strategies using the community networks described above, disease transmission is simulated using each network. The Adaptive Recovery Model developed at Sandia National Laboratories [12] was used to simulate disease transmission with various vaccination strategies. This model builds on the software developed by McGee [16], which integrates network structure into a stochastic compartment model. The model includes compartments that represent susceptible (S), exposed (E), asymptotic (A), infectious (I), hospitalized (H) recovered (R), and dead (D) states. The transmission paths between states are shown in Fig. 5. While additional modeling options have been included to model the impact of quarantine, contact tracing, and surveillance sampling, those options are not used in this analysis.

**Table 3:** Disease transmission parameters with age dependent values based on [26]

Parameter	Value
Transmission probability, $\beta$	0.08
Fraction that remain asymptomatic, $f_A$	0.3
Fraction that are hospitalized, $f_H$	0.04
Fraction that recovers, $f_R$	Age < 18: 0.9999
	18 $>=$ Age < 50: 0.9995
	50 $>=$ Age < 65: 0.9940
	Age $>=$ 65: 0.9100
Time from exposed to asymptomatic, $T_E$	4 days
Time from asymptomatic to infected, $T_A$	6 days
Time from infected to hospital, $T_I$	Age < 18: 2 days
	18 $>=$ Age < 50: 6 days
	50 $>=$ Age < 65: 6 days
	Age $>=$ 65: 4 days
Time spent in the hospital, $T_H$	Age < 18: 3.5 days
	18 $>=$ Age < 50: 6.5 days
	50 $>=$ Age < 65: 9 days
	Age $>=$ 65: 9 days
Time from recovered to susceptible, $T_R$	36500 days

The disease transmission parameters used in this analysis are shown in Table 3. Transition times between disease states are based on CDC planning scenarios which define some parameters as a function of age [26]. Age dependent disease progression is an important factor in the study of vaccine strategies, as older individuals have a higher chance of hospitalization and death and therefore benefit from the vaccine more. Note that the time from R state to S state is set to 100 years ($T_R$ in Table 3). This means that individuals are rarely reinfected. In reality, this is not true. However, this focuses the analysis on the use of vaccines to reduce first infections. The impact of vaccine strategies on subsequent infections could be included in future analysis.

The community networks are initialized with 0.56% of the population in the I state, 0.02% of the population in the H state, and 6.07% of the population in the R state. These values were derived from data obtained from a COVID-19 tracking dashboard in 2021 for Bernalillo County in New Mexico. Using the cumulative cases, hospitalizations, and recovery counts a relative seroprevalence was estimated to initialize the analysis. Each scenario is simulated for 365 days, or until there is no longer disease within the community. It is important to keep in mind that the disease transmission model has not been calibrated to the communities of interest. Rather, the disease transmission parameters and differences in community structure provide a basis for comparing the relative impact of disease control strategies.

To model the impact of vaccine strategies, individual vaccine status (unvaccinated, partially vaccinated, fully vaccinated) is tracked per network node, where each node represents an individual. A two dose vaccine is modeled in this analysis. The time between doses is set to 3 weeks. The vaccine efficacy is based on the Pfizer vaccine clinical trials (0.82 after the first dose and 0.94 after the second dose) [27]. Vaccination reduces infectiousness by increasing the likelihood that an individual remains asymptomatic (A state). This is nominally set to 0.3 ($f_A$ in Table 3). Vaccines are distributed based on the available stock, queue of eligible people, and a prioritization strategy.

Vaccine availability is defined as the percent of the population that could be vaccinated each week, based on available stock. For this analysis, it is assumed that vaccine availability is held constant over the simulation time frame. For example, a peak of 3.38 million doses per day were administered in the U.S. on April 13, 2020 [28]. If this peak rate was sustained, that is equivalent to a vaccine availability of 7.2% of the population each week.

Individuals are vaccine eligible if they are in the S, E, A, or R state. As such, a queue of eligible people changes over time, as a function of disease transmission and vaccine status. Therefore, it is possible for someone to receive a first does and then become ineligible for a second dose if the individual becomes symptomatic (I state) within the three weeks between doses. In situations where resources are limited (the length of the queue is greater than the available vaccine doses for a particular day), only the highest-weighted individuals are selected for vaccination. Those that require a second dose are moved to the top of the queue. Additionally, children under 5 were not eligible for vaccination in the U.S until very recently. Given that the closest age bin in this analysis is 10, anyone under 10 is included as ineligible in this analysis. As mentioned earlier, the time spent in R is very large in this analysis, and therefore very few people transition back into the S state. In that way, vaccines given to people in the R state have very little impact on disease control.

People that are not willing to be vaccinated are removed from the vaccine queue. The fraction of the population willing to receive a vaccine has changed over the course of the pandemic. As of February 2022, 65% of the U.S. population was fully vaccinated [28]. Future research could include vaccine willingness that is a function of age, other demographics, or disease incidence in the population.

Position in the queue is determined by different factors according to a vaccine strategy. In this analysis, factors include age, household size, node degree, and disease state of neighboring nodes (for ring vaccination). Age vaccination sorts the queue based on age, from oldest to youngest. Household size vaccination sorts the queue based on household size, from large households to small households. Node degree vaccination sorts the queue based on the number of contacts a person has per day, from largest to smallest. Based on the way the network is generated, the number of contacts a person has per day is the node degree. Ring vaccination gives priority to people that are connected to someone that became symptomatic (transitioned into the I state). The list of people associated with this “ring” is updated every time step. This assumes the time associated with contact tracing is very small. Random vaccination, with no prioritization, is also included in the study. While information on age and household size is easy to obtain within a population, the number of contacts and the disease state of contacts is less observable. Community-aware centrality measures have also been studied as a way to prioritize immunization to reduce the size of disease outbreaks [29–31]. While centrality is also hard to observe in communities, the framework presented here could be extended to study the impact of vaccine prioritization based on centrality. Additional vaccine strategies, such as occupation and heath status could also be tested within this modeling framework, but would require additional data that is not currently included in this study.

To include the influence of non-pharmaceutical control measures, the use of personal protective equipment in the form of masks is included in the analysis. Masks can offer a wide range of protection, depending largely on the material and how the mask is worn. In this analysis, mask effectiveness is defined as the product of the probability that an individual wears a mask and the protection that the mask offers. For example, if an individual wears a mask 25% of the time and the mask reduces transmission by 50%, then the mask effectiveness is 0.125. In the disease transmission model, mask effectiveness is used to modify the transmission probability such that $\widetilde{\beta } = \beta *(1-m)$ where $\widetilde{\beta }$ is the modified transmission probability, $\beta$ is the original transmission probability (set to 0.08 in this analysis) and *m* is the mask effectiveness. Masks are assumed to only be used outside of households. Therefore, transmission probability is only modified when people interact with members of different households, as defined by the network structure. While additional non-pharmaceutical control measures could be included in the analysis, including quarantine and contact tracing, the use of these control measures has declined in most communities.

### Global sensitivity analysis

**Table 4:** Variables used in the global sensitivity analysis

Variable	Type	Values
Community	Categorical	High Age, High Population, High SVI, High Household Size
Vaccine prioritization	Categorical	Random, Age, Node degree, Household size, Ring
Vaccine willing	Continuous	Uniform[0.1, 0.9]
Mask effectiveness	Continuous	Uniform[0, 0.8]
Random seed	Discrete	$[0,9]\cap {\mathbb {N}}$

Global sensitivity analysis (GSA) apportions the influence that model parameter uncertainties have on the uncertainty of model output [32]. In this analysis, GSA is used to evaluate the influence that vaccine willingness and mask effectiveness have on disease outcomes across the four distinct communities (High Age, High Population, High SVI, High Household Size) and five distinct vaccine strategies (Random, Age, Node degree, Household Size, and Ring). Table 4 summarizes the input variables utilized in the sensitivity analysis.

The fraction of the population willing to be vaccinated is defined using a uniform distribution over the interval [0.1, 0.9]. This assumes that at least 10% of the population is willing and at least 10% is not willing. Mask effectiveness is defined using a uniform distribution over the interval [0, 0.8]. The lower bound reflects a case where masks are no longer in use. The upper bound defines a high quality mask worn correctly most of the time. The random seed controls the network structure, as described above, along with the initialized state of each person and the stochastic process of selecting transitions between states. Ten random seeds were used in this analysis. Additional parameters that control vaccine administration were held constant. This includes vaccine availability and vaccine efficacy. Vaccine availability maintains a vaccine supply that cover 5% of population each week. To account for real-world conditions, vaccine efficacy from clinical trials was reduced by 20%. As such, the first vaccine dose provides an efficacy of 66% and the second dose provides an efficacy of 75%.

Several quantities of interest were derived from the simulation results to evaluate the impact of vaccination on the population. This includes peak hospitalization, cumulative disease related deaths, and years of life lost (YLL). Peak hospitalization is defined as *max*(*H*(*t*)) for $t\in [t_0,t_f]$ where $t_0$ is the start of the simulation at day 0 and $t_f$ is the end of the simulation at 365 days (or until there is no longer disease within the community). Cumulative deaths is $D(t_f)$ (total deaths at the end of the simulation). YLL uses the expectation of life at a specific age from the World Health Organization [33]. YLL is computed as $YLL = \sum _{n} D_n(t_f)*\mathbb {E}[X_n]$ where n is an age bin, $D_n(t_f)$ are the cumulative deaths in that age bin, and $\mathbb {E}[X_n]$ is the expectation of life for that age.

Stochastic models of chaotic systems requires careful consideration for numerically deriving the sensitivity indices of variance-based decompositions methods such as Sobol’ indices [34]. Classical GSA is theoretically grounded analysis when variability inherent to model input is governed by continuous random variables. In the event that the source of uncertainty is derived from discrete random variables it is still plausible to design a GSA [35, 36], but not advisable when the discrete random variable is inherent to the instantiation of stochastic network topologies. To obtain numerical stability with regards to GSA of stochastic models, Hart et al. [34] have shown that partitioning the influence of uncertainty inherent to the randomized algorithm from parameter uncertainty is essential. Therefore a full GSA is run for each random seed, resulting in variability of results for the Sobol’ indices. Additionally, the four distinct communities and the five different vaccination strategies represent additional discrete random variables of interest. It is important to make the clarification that these random variables have categorical random variates that do not have a measurable relationship on their space of realizations, resulting in even another layer of complexity to the GSA. Thus, the variability of GSA results is extended beyond the random initialization of the network to also consider the whole combinatorial space defined by 10 random seeds, 4 communities, and the 5 vaccine prioritization strategies.

To support efficient numerically approximation for Sobol’ indices, Saltelli sampling [37] was implemented using the Python package SALib [38]. The Saltelli sample was derived from the continuous input variables in Table 4, vaccine willingness and mask effectiveness. To explore first-order and second-order sensitivity, the recommended $N*(2*D+2)$ sample vectors was collected, where *N* is the number of unique values for each input variable (generally a power of 2) and *D* is number of input variables. In this analysis, D is 2 and N was set to $2^{8}$, resulting in a total of 1,536 samples drawn from uniform distributions for each variable. The Saltelli sample is then reused for each combination of categorical and discrete variables: 5 communities, 4 vaccine strategies, and 10 random seeds. This results in $200*1,536 = 307,200$ simulations of the stochastic network model.

Since the goal of this analysis is to understand the effects that variability in vaccine willingness and mask effectiveness has on peak hospitalization, cumulative deaths, and YLL, the variability of network topology was restricted to 10 random seeds for each of the 20 combinations of communities and vaccine strategies. For proper treatment of the uncertainty of the Sobol’ indices, orders of magnitude more random variates are required to statistically represent the uncertainty inherent to the network topologies. As a qualification check on the results, 10 different seeds were run while the rest of the experimental design was held constant. The results of the qualification check were consistent with the original analysis provided in the following section.

## Results

The modeling framework described above provides a basis to study the influence of vaccine prioritization strategies on communities that have different demographics and connectivity. In this analysis, the vaccine prioritization strategies include vaccines prioritized by age, node degree, household size, and ring vaccination. The study also includes cases where people who are willing and eligible are vaccinated at random. Masking is included as a non-pharmaceutical control measure. The following section describes results from the analysis.

**Fig. 6:**
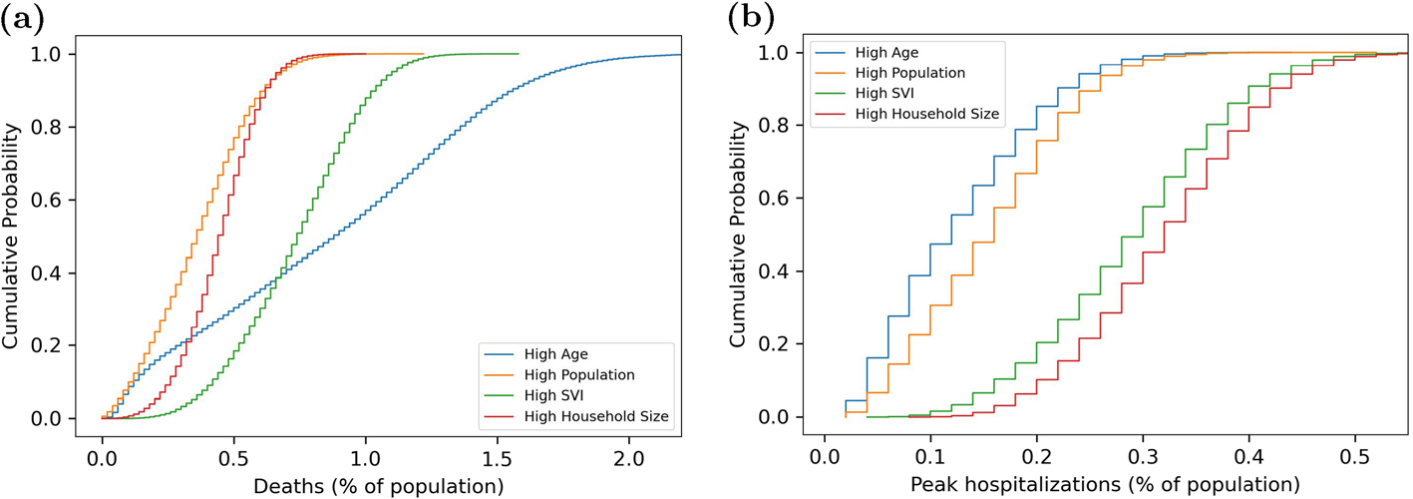
Empirical cumulative distribution of **a** deaths and **b** peak hospitalization for each community

Figure 6 shows the distribution of deaths and peak hospitalization for each community. Each distribution takes into account variability in vaccine strategy, vaccine willingness, mask effectiveness, and the random seed. As shown in the appendix, YLL is very similar to deaths given that most deaths occur within populations above 65 years of age. For that reason, this paper focus on deaths and peak hospitalization.

**Fig. 7:**
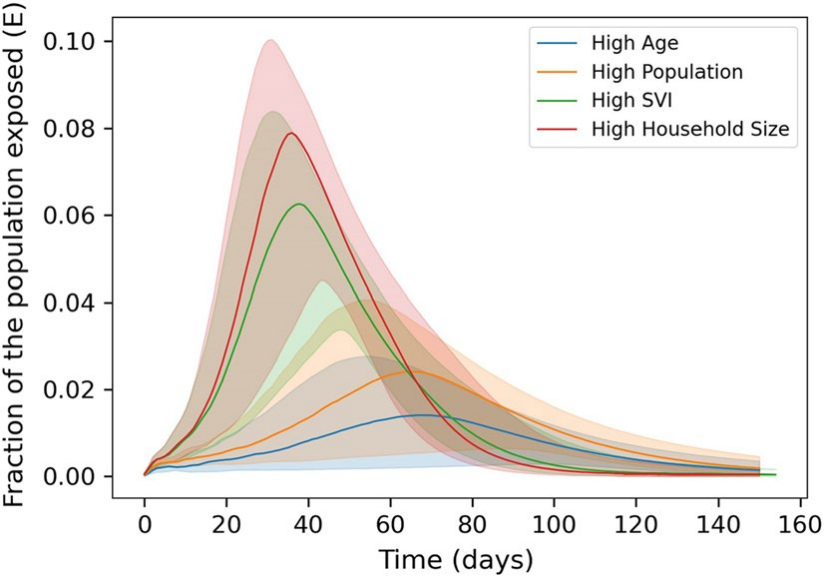
Fraction of the population exposed as a function of time. Plot includes the median (line) and standard deviation (shaded region) for each community from the full set of simulations

As expected, significant differences in deaths and peak hospitalization are seen across communities. The rate at which disease is transmitted within each community is a large contributing factor, as shown in Fig. 7. Peak hospitalizations are generally higher and occur earlier in the High SVI and High Household Size communities. These communities have relatively high node degree and clustering and relatively high number of contacts per day among older people. Note that the timing of peak hospitalization (at approximately 40 days) is roughly two and a half weeks after members of the population could become fully vaccinated (21 days). This means that even if vaccines are distributed as quickly as possible, vaccination will have limited impact on peak hospitalization in these highly connected communities. On the other hand, deaths are highest in the more isolated High Age communities. These communities have lower node degree and clustering, along with a high mortality risk. The SVI communities also have relatively high deaths. These communities have relatively high age and high number of contacts per day among older populations. It is important to recognize that all communities include cases where disease transmission resulted in very low deaths and peak hospitalizations. This can occurs when mask effectiveness is very high, vaccine willingness is very high, or the initialized case load is in places where network connectivity, and therefore initial disease transmission, is very low.

### Sensitivity indices

Using GSA, variability in the model output is decomposed into fractions that are attributed to each model input variable. The model output variance is quantified using deaths and peak hospitalization and model input variables include vaccine willingness and mask effectiveness. This process is repeated for each of the 200 combinations of community, vaccine prioritization strategy, and random seed. For each metric (deaths and peak hospitalization), this results in 200 values for first-order sensitivity (or main effects) for each input variable and 200 values for second-order sensitivity for each combination of input variables. While the same analysis was carried out for each metric, the figures and discussion below focus on cumulative deaths. Results from the sensitivity analysis using peak hospitalization are shown in the appendix. Results using peak hospitalization reveals that mask effectiveness is highly influential across all communities and vaccine prioritization strategies. As mentioned earlier, disease exposure peaks a few weeks after people can become fully vaccinated. As such, vaccine willingness has a lesser impact on peak hospitalization.

**Fig. 8:**
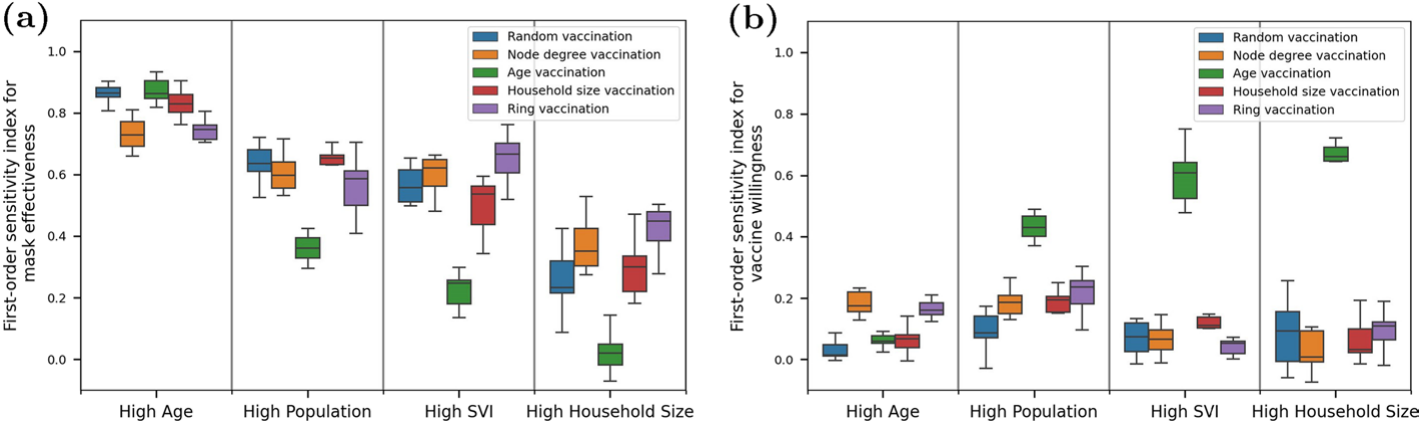
Distribution of first-order sensitivity for **a** mass effectiveness and **b** vaccine willingness on deaths. Results are grouped by community and vaccine prioritization. Each box includes the 25th, 50th and 75th percentile for the model output, with whiskers that extend to 1.5 times the interquartile range

Figure 8 shows the distribution of first-order sensitivity on deaths. The results are grouped by community and vaccine prioritization strategy. Each bar includes variability associated with the random seed. The analysis indicates that sensitivity of mask effectiveness on deaths decreases as communities are more connected (Fig. 8a). While the High Age community is very sensitive to mask effectiveness, the High Household Size community is not. Since it is assumed that people do not wear masks inside households, masking is a less effective control measure in these communities. This trend is especially pronounced when age vaccination is used. When older individuals are vaccinated first in highly connected communities, deaths are less sensitive to masking. In general, vaccine willingness has a lesser impact on deaths (Fig. 8b). Especially in more isolated High Age communities, deaths are not very sensitive to vaccine willingness where the immediate effect of masking can be more impactful. However, communities that are more connected are sensitive to vaccine willingness when using age prioritization. In these cases, deaths are more sensitive to vaccine willingness as long as older individuals are vaccinated first. This can impact disease related outcomes on the rest of the highly connected community.

**Fig. 9:**
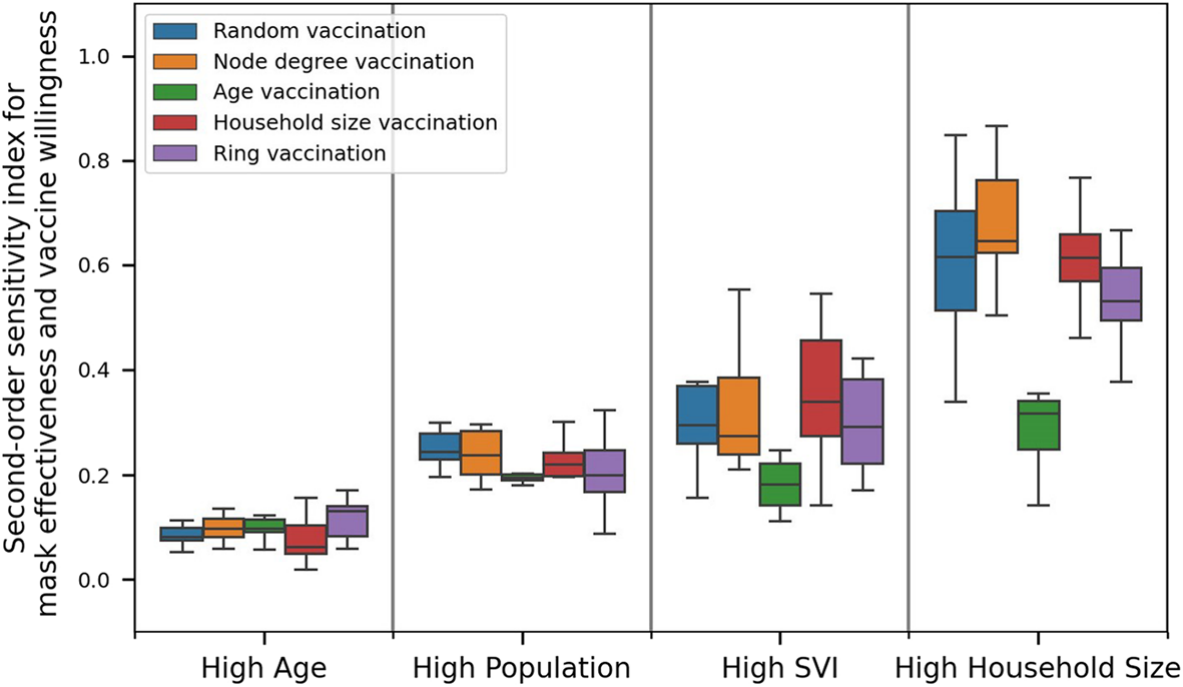
Distribution of second-order sensitivity on deaths. Results are grouped by community and vaccine prioritization. Each box includes the 25th, 50th and 75th percentile for the model output, with whiskers that extend to 1.5 times the interquartile range

Figure 9 shows the distribution of second-order sensitivity on deaths. Results indicate increasing second-order effects for communities that are more connected. Within highly connected communities, the combined influence of masking and vaccination is required to control deaths, especially when vaccine strategies other than age prioritization are used. In contrast, second-order sensitivity are small in the more isolated High Age population, where most of the sensitivity is explained by mask effectiveness.

In this analysis, the random seed accounts for network structure and initialization. The stochastic nature of this process results in more variability when communities are highly connected. When networks are heterogeneous, the nature of interactions within and between clusters and knowing disease state are critical aspects to control transmission. When networks are more uniform and isolated, understanding the exact nature of these interactions becomes less important.

### Scenario analysis

To further investigate the influence of vaccine strategies and masking on disease outcomes, the following analysis looks at two scenarios to identify trends. The scenario analysis compliments the GSA by illustrating how changes in mask effectiveness and vaccine willingness can drive down deaths and peak hospitalizations. The scenarios isolate conditions in which vaccine strategies could have an impact on disease outcomes. Each scenario is defined using subsets of model input variables, as defined below:

- Scenario 1: Vaccine willingness is moderately high (between 0.6 and 0.8), mask effectiveness varies from low to high (between 0 and 0.8).
- Scenario 2: Mask effectiveness is low (between 0 and 0.2), vaccine willingness varies from low to high (between 0.1 and 0.9).

To illustrate trends and uncertainty, the simulation results from each scenario are grouped into 10 equally spaced bins with respect to mask effectiveness for Scenario 1 and vaccine willingness for Scenario 2. For each community, vaccine prioritization strategy, and metric (deaths and peak hospitalization) the average and standard deviation is computed and used to visualize results. While the trends could be explored using linear or higher order regression, the binned approach captures important non-linearity in the simulation results.

**Fig. 10:**
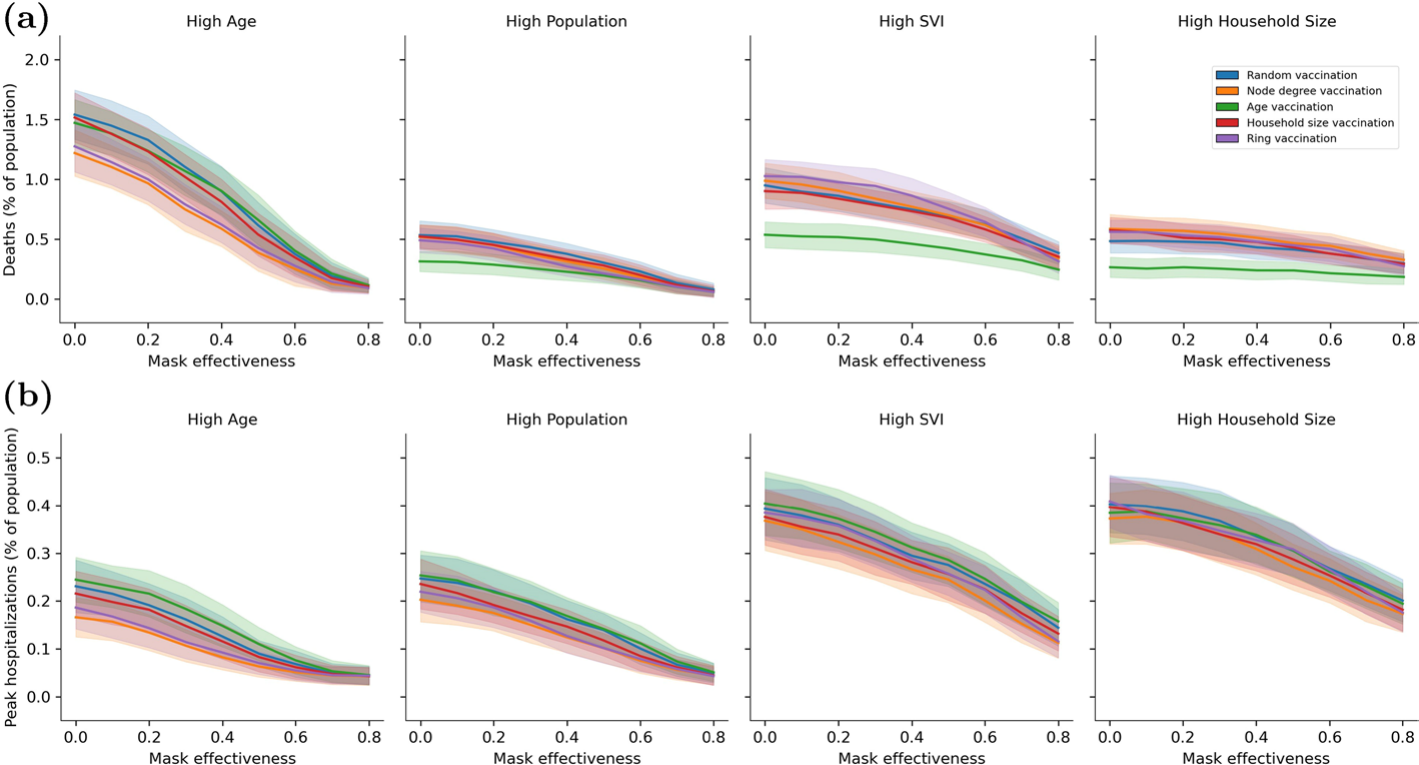
Impact of mask effectiveness and vaccine strategies on **a** deaths and **b** peak hospitalization using Scenario 1 where vaccine willingness is moderately high

Results from Scenario 1 are shown in Fig. 10. Figure 10a uses deaths and Fig. 10b uses peak hospitalization. There is one subplot per community and each subplot includes results from all 5 vaccine strategies. The results show that a high level of mask effectiveness can drive down deaths and peak hospitalization to very low levels. However high level of mask use is rarely sustained in practice. As mask effectiveness decreases, selecting a specific vaccine prioritization strategy can provide significant benefit. This is most pronounced in the High Population, High SVI, and High Household Size communities where age vaccine prioritization performs best when the objective is to reduce deaths. As compared to other vaccine strategies, the benefit to using age vaccine prioritization can be as high as 0.4% (based on the average from each strategy). In the High Age communities, node degree and ring vaccine prioritization perform slightly better at reducing deaths and reducing peak hospitalization. There is less difference between vaccination prioritization with respect to peak hospitalization in other communities.

**Fig. 11:**
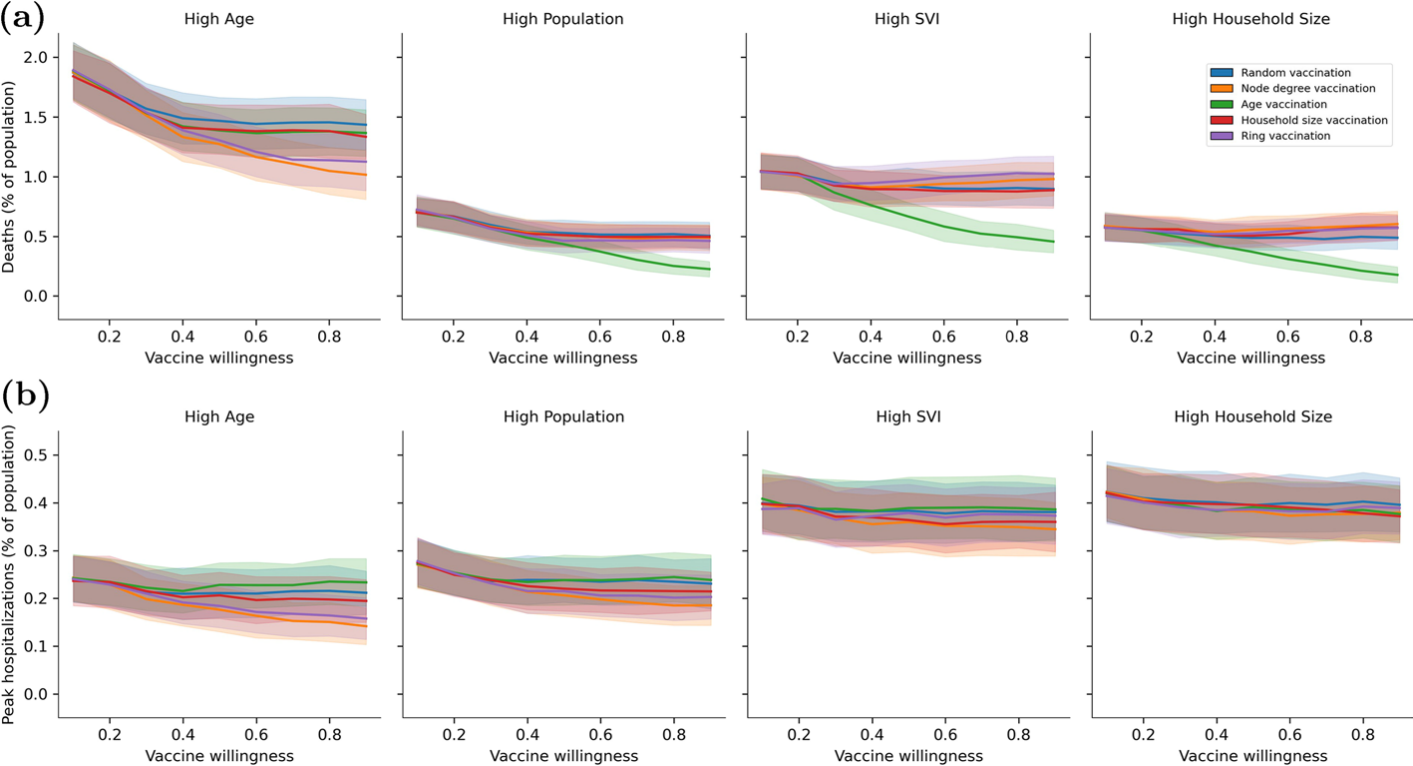
Impact of vaccine willingness and vaccine strategies on **a** deaths and **b** peak hospitalization using Scenario 2 where mask effectiveness is low

Results from Scenario 2 are shown in Fig. 11. Figure 11a uses deaths and Fig. 11b uses peak hospitalization. As with the previous figure, there is one subplot per community and each subplot includes results from all 5 vaccine strategies. The results show that as vaccine willingness increases, selecting a specific vaccine prioritization strategy can provide significant benefit. This is most pronounced when the objective is to reduce deaths. In the High Age community, node degree and ring vaccine prioritization provide significant benefit, whereas in the other communities age vaccine prioritization is the most beneficial. The difference in outcomes when using different vaccine strategies can be as high as 0.5% (based on the average from each strategy). Interestingly, all communities hit an asymptote with respect to deaths when using certain vaccine prioritization strategies. For example, the use of random, household size prioritization, and age prioritization in the High Age community does not continue to decline after willingness reaches approximately 50% of the population. Similar thresholds are noted in other communities. Certain vaccine prioritization strategies continue to drive down deaths as the population is more willing to be vaccinated. However, the strategy is not the same for each community. For example, there is an advantage to using node degree and ring vaccination in the High Age community if a high percent of the population is willing to be vaccinated. In this case, targeting individuals who contribute to transmission outweighs targeting individuals by age (and therefore higher mortality risk). In the High Population, High SVI, and High Household Size communities, the age prioritization strategy continues to outperform other vaccine prioritization as more people are willing to be vaccinated. These differences are a function of community structure. When the objective is to reduce peak hospitalization, the differences between vaccine strategies is less pronounced.

The scenarios demonstrate that under certain conditions vaccine prioritization strategies can reduce deaths and peak hospitalization. However, vaccination prioritization strategies do not always outperform random vaccination. The results presented here indicate that the structure and demographics of a community should play an important role in determining if prioritization strategies should be applied to achieve a desired objective.

## Conclusion

Prioritizing vaccine administration was an urgent concern when COVID-19 vaccines first became available. Protecting people most likely to suffer severe outcomes and people that are essential for healthcare and other services motivated allocation of initial doses. With increased availability of vaccines in the U.S. and other developed countries, prioritization is a less pressing concern. However, understanding of the differential epidemiological effects of different vaccine strategies remains useful. New variants and loss of immunity may recapitulate the initial phase of the pandemic, leading to scarcity in effective control measures, and the need to carefully allocate new vaccines, and motivate their uptake to insure maximum benefit. Additionally, many countries still face limited vaccine supply and might benefit from insights into the effects of different priorities. A comparative assessment of vaccine strategy performance, particularly one that considers different community compositions and interaction patterns, can therefore provide useful guidance to decision makers when vaccination is impeded by constraints on either supply or demand.

This research outlines a modeling framework that explores the impact of vaccine prioritization based on community characteristics. Results from this analysis highlights significant differences in vaccine strategies for communities that have different demographics and structure. The results also show that age vaccine prioritization does not always result in the best outcomes, especially in High Age communities. Furthermore, in some settings, random vaccination can perform as well as more carefully planned strategies. This is important to keep in mind when strategies require additional resources, such as contact tracing for ring vaccination. The results also illustrate that heterogeneous network models are an effective tool for capturing the effects of interactions at the household level. This is especially important when modeling communities that have a high degree of clustering and when modeling behavior that is not uniformly applied (for example, not wearing masks inside the home). Understanding the complex interactions between community structure, mask use, and vaccination could lead to more informed decisions for vaccine prioritization.

## Figures and Tables

**Fig. 12:**
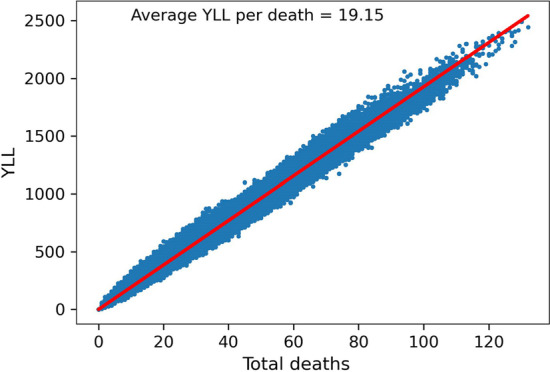
Scatter plot comparing deaths with YLL for each simulation in the analysis (blue dots) along with a best fit line (red) that has a slope of 19.15

**Fig. 13:**
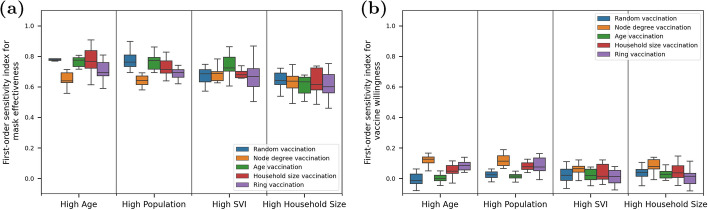
Distribution of first-order sensitivity for **a** mass effectiveness and **b** vaccine willingness on peak hospitalizations. Results are grouped by community and vaccine prioritization. Each box includes the 25th, 50th and 75th percentile for the model output, with whiskers that extend to 1.5 times the interquartile range

**Fig. 14:**
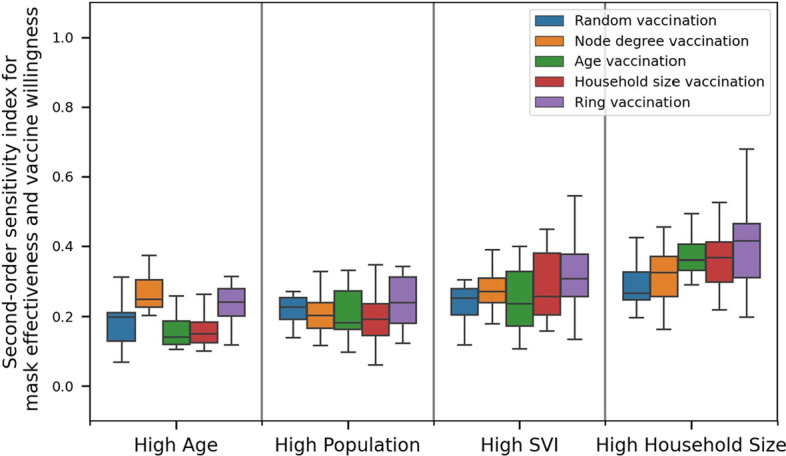
Distribution of second-order sensitivity on peak hospitalizations. Results are grouped by community and vaccine prioritization. Each box includes the 25th, 50th and 75th percentile for the model output, with whiskers that extend to 1.5 times the interquartile range

## Data Availability

The datasets used and/or analysed during the current study are available from the corresponding author on reasonable request.

## Appendix

The following appendix includes 1) the relationship between deaths and YLL based on the simulations run in the global sensitivity analysis and 2) results from the global sensitivity analysis using peak hospitalization.

Figure 12 shows the relationship between deaths and YLL. The slope of the best fit line results in an average YLL of 19.15 years which is very similar to the expectation of life for a person 65 years old. While the simulations include deaths across all age bins, the high correlation between deaths and YLL means that investigating vaccine strategies using deaths and YLL produce similar results.

Figures 13 and 14 show first and second-order sensitivity indices for peak hospitalization. Results show that mask effectiveness is the primary driver for sensitivity across all communities and vaccine prioritization strategies. Vaccine willingness has a lesser impact on peak hospitalization. As communities are more connected, there is a slight increase in second-order sensitivities, indicating that combined masking and vaccinations impact results.
